# Nongenomic Glucocorticoid Suppression of a Postsynaptic Potassium Current via Emergent Autocrine Endocannabinoid Signaling in Hypothalamic Neuroendocrine Cells following Chronic Dehydration

**DOI:** 10.1523/ENEURO.0216-17.2017

**Published:** 2017-09-19

**Authors:** Ning Wu, Jeffrey G. Tasker

**Affiliations:** 1 Department of Cell & Molecular Biology, Tulane University, New Orleans, LA 70117; 2 Tulane Brain Institute, Tulane University, New Orleans, LA 70118

**Keywords:** corticosteroid, HPA, magnocellular, oxytocin, stress, vasopressin

## Abstract

Glucocorticoids rapidly stimulate endocannabinoid synthesis and modulation of synaptic transmission in hypothalamic neuroendocrine cells via a nongenomic signaling mechanism. The endocannabinoid actions are synapse-constrained by astrocyte restriction of extracellular spatial domains. Exogenous cannabinoids have been shown to modulate postsynaptic potassium currents, including the A-type potassium current (*I*_A_), in different cell types. The activity of magnocellular neuroendocrine cells is shaped by a prominent *I*_A_. We tested for a rapid glucocorticoid modulation of the postsynaptic *I*_K_ and *I*_A_ in magnocellular neuroendocrine cells of the hypothalamic paraventricular nucleus (PVN) using whole-cell recordings in rat brain slices. Application of the synthetic glucocorticoid dexamethasone (Dex) had no rapid effect on the *I*_K_ or *I*_A_ amplitude, voltage dependence, or kinetics in magnocellular neurons in slices from untreated rats. In magnocellular neurons from salt-loaded rats, however, Dex application caused a rapid suppression of the *I*_A_ and a depolarizing shift in *I*_A_ voltage dependence. Exogenously applied endocannabinoids mimicked the rapid Dex modulation of the *I*_A_, and CB1 receptor antagonists and agonists blocked and occluded the Dex-induced changes in the *I*_A_, respectively, suggesting an endocannabinoid dependence of the rapid glucocorticoid effect. Preincubation of control slices in a gliotoxin resulted in the partial recapitulation of the glucocorticoid-induced rapid suppression of the *I*_A_. These findings demonstrate a glucocorticoid suppression of the postsynaptic *I*_A_ in PVN magnocellular neurons via an autocrine endocannabinoid-dependent mechanism following chronic dehydration, and suggest a possible role for astrocytes in the control of the autocrine endocannabinoid actions.

## Visual Abstract

**Figure:**
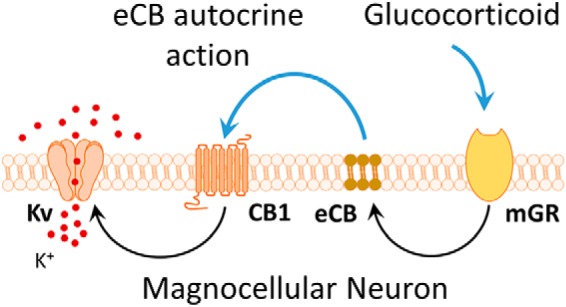

## Significance Statement

Stress causes elevated levels of glucocorticoid hormones and rapid and delayed glucocorticoid feedback effects in the brain. Glucocorticoids regulate synaptic inputs to hypothalamic neuroendocrine cells via a nongenomic release of endocannabinoid. We report a nongenomic glucocorticoid modulation of a postsynaptic A-type potassium current in magnocellular neurons via a novel autocrine endocannabinoid mechanism. The A-current modulation by glucocorticoids occurred in neurons from rats subjected to chronic dehydration via salt loading, but not in neurons from normally hydrated rats. Our findings suggest that chronic dehydration leads to glucocorticoid-induced endocannabinoid autocrine signaling in magnocellular neuroendocrine cells. The neuroplastic mechanisms for this emergent signaling may be related to neuronal–glial structural plasticity or to changes in rapid postsynaptic glucocorticoid and/or endocannabinoid actions.

## Introduction

Osmotic challenge elicits a neuroendocrine stress response that results in an increase in the circulating level of glucocorticoids (Roberts et al., 2011). Stress levels of glucocorticoids cause endocannabinoid synthesis in paraventricular nucleus (PVN) magnocellular neuroendocrine cells (MNCs) and parvocellular neuroendocrine cells (Di et al., 2003, 2005a, 2009; Malcher-Lopes et al., 2006). Endocannabinoids are synthesized from lipid precursors in neuronal membranes and are released canonically as retrograde signals to regulate the presynaptic release of glutamate and GABA (Di Marzo, 2011). Endocannabinoid synthesis is elicited in magnocellular neuroendocrine cells of the hypothalamus by depolarization (Hirasawa et al., 2004; Di et al., 2005b) and in response to oxytocin (Oliet et al., 2007), as well as in response to rapid glucocorticoid actions (Di et al., 2003, 2005a). Glucocorticoid- and depolarization-induced retrograde endocannabinoid release from MNCs of the hypothalamic PVN and supraoptic nucleus (SON) causes a synapse-specific suppression of glutamate release at excitatory synapses (Tasker, 2006; Tasker et al., 2006). Restriction of the retrograde endocannabinoid actions to glutamate synapses is controlled by astrocytes, since salt loading-induced neuronal–glial plasticity and pharmacologic inhibition of glial cell metabolism lead to spillover actions of endocannabinoids at neighboring GABA synapses (Di et al., 2013).

While endocannabinoids have been identified predominantly as retrograde messengers that regulate presynaptic neurotransmitter release, exogenous cannabinoids also have been reported to modulate postsynaptic potassium channels (Deadwyler et al., 1995; Mackie et al., 1995; Schweitzer, 2000). Glucocorticoids also have been shown to rapidly regulate postsynaptic properties by inhibiting potassium currents in PVN neurons (Zaki and Barrett-Jolley, 2002) and hippocampal neurons (ffrench-Mullen, 1995; Olijslagers et al., 2008) and by modulating calcium-dependent potassium channels in pituitary GH3 and AtT-20 cells (Huang et al., 2006). The mechanisms of these postsynaptic actions of glucocorticoids have not been characterized.

The A-type potassium current (*I*_A_) is a prominent voltage-gated conductance in PVN magnocellular neurons that influences magnocellular neuron firing properties (Luther and Tasker, 2000; Ellis et al., 2007). Water deprivation or chronic salt loading induces dramatic structural and functional plasticity among magnocellular neuroendocrine cells and astrocytes of the hypothalamic PVN and supraoptic nucleus (Tasker et al., 2017). This plasticity results in functional changes in magnocellular neuron electrical activity that lead generally to an increase in excitability (Tasker et al., 2002). Here, we investigated whether glucocorticoids rapidly modulate the *I*_K_ and *I*_A_ of magnocellular neurons in a transcription-independent fashion via autocrine endocannabinoid signaling, and whether the rapid glucocorticoid modulation of the *I*_K_ and *I*_A_ is altered with chronic osmotic stress via salt loading.

## Materials and Methods

### Animals

Male Sprague Dawley rats (5–6 weeks old; Harlan Laboratories) were used with the approval of the Tulane University Animal Care and Use Committee and in accordance with US Public Health Service guidelines. Rats were dehydrated via chronic salt loading by restricting them to drinking water with 2% NaCl for 6–8 d. Untreated control rats were age matched and were provided with regular tap drinking water.

### Hypothalamic slice preparation

Rats were decapitated with a rodent guillotine under deep halothane anesthesia. The brain was quickly removed and placed into ice-cold artificial CSF (aCSF) containing the following (in mm): 140 NaCl, 3 KCl, 1.3 MgSO_4_, 1.4 NaH_2_PO_4_, 2.4 CaCl_2_, 11 glucose, and 5 HEPES, with pH adjusted to 7.2–7.3 with NaOH, the osmolarity was 290–300 mOsm, and the aCSF was bubbled with 100% O_2_. The hypothalamus was blocked and the caudal surface of the tissue block was glued to the chuck of a vibrating tissue slicer. Two coronal hypothalamic slices 300 µm in thickness and containing the PVN were sectioned, bisected at the midline, and submerged in a holding chamber in oxygenated aCSF at room temperature, where they were allowed to equilibrate for >1.5 h before being transferred to a recording chamber.

### Electrophysiology

Patch pipettes were pulled from borosilicate glass with a Flaming/Brown P-97 micropipette puller (Sutter Instruments) to a tip resistance of 3–4 MΩ. They were filled with a solution containing the following (in mm): 120 K-gluconate, 10 KCl, 1 NaCl, 1 MgCl_2_, 1 EGTA, 2 Mg-ATP, 0.3 Na-GTP, and 10 HEPES; the pH was adjusted to 7.2 with KOH, and the osmolarity was adjusted to 300 mOsm with 20 mm d-sorbitol. For recordings, single hemi-slices were transferred from the holding chamber to a submersion recording chamber, where they were perfused with oxygenated aCSF at a rate of 2 ml/min and allowed to equilibrate for at least 15 min before starting the recordings. PVN neurons were visualized on a video monitor with a cooled CCD camera using infrared illumination and differential interference contrast optics, and were patch clamped under visual control. After achieving the whole-cell configuration, the series resistance was compensated by ≥60% and the series resistance and whole-cell capacitance were continuously monitored during experiments. Magnocellular neurons in the PVN were identified based on their location, size, morphology, and electrophysiological properties (i.e., prominent *I*_A_ in voltage clamp and transient outward rectification in current clamp; Tasker and Dudek, 1991; Luther and Tasker, 2000). Recordings were performed using a Multiclamp 700A amplifier (Molecular Devices). Data were low-pass filtered at 2 kHz with the amplifier and sampled at 10 kHz using the pClamp 9 data acquisition and analysis software package (Molecular Devices).

Before experiments, cells were tested in current-clamp mode and excluded from analyses if they did not meet the following criteria: action potential amplitudes ≥50 mV from the threshold to the peak, an input resistance at resting potential of at least 500 MΩ, a resting membrane potential negative to −50 mV, and a characteristic transient outward rectification (Tasker and Dudek, 1991; Luther and Tasker, 2000). Slices were then bathed in aCSF with tetrodotoxin (TTX; 1 µm) and CdCl_2_ (200 µm) to block voltage-gated sodium channels and calcium channels, respectively, and the recording configuration was switched to voltage clamp. The liquid junction potential (calculated at 15 mV) was corrected *post hoc* during data analysis. All voltage-clamp recordings were leak subtracted using a P/4 protocol. Series resistance compensation of ≥60% was routinely applied, and changes in series resistance were monitored and compensated for throughout the experiments. The series resistance at the beginning of recordings was <20 MΩ, and recordings were discarded if changes in series resistance of >20% occurred during the recordings.

### Voltage dependence of activation and inactivation of the *I*_A_ and *I*_K_

**Figure 1.**
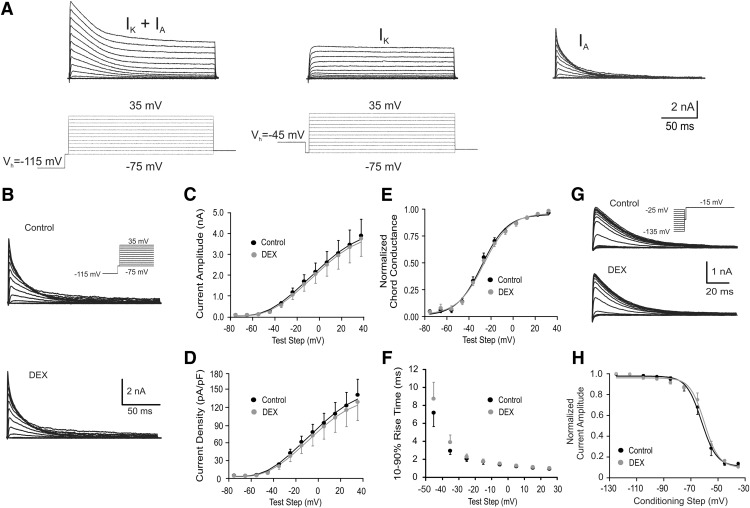
Dexamethasone had no rapid effect on *I*_A_ activation and inactivation in magnocellular neurons from untreated rats. ***A***, Voltage-clamp protocol for isolating *I*_K_ and *I*_A_. Combined *I*_K_ and *I*_A_ currents were evoked by stepping from a holding potential (*V*_h_) of −115 mV to test steps of −75 to 35 mV in 10 mV increments (left: voltage steps below, current responses above). *I*_K_ was evoked by the same stepping protocol, except from a *V*_h_ of −45 mV (middle: voltage steps below, current responses above). Subtraction of the currents activated from a *V*_h_ of −45 mV from those activated from a *V*_h_ of −115 mV yielded the isolated low voltage-activated *I*_A_ (right current traces). ***B***, Representative traces showing voltage-dependent activation of *I*_A_ in PVN magnocellular neurons from untreated rats before and at the end of a 10 min Dex application (1 µm). Inset, Activation voltage-clamp protocol. ***C***, Plots of the mean current amplitude of the *I*_A_ vs the test step potential, showing no effect of Dex on the *I*_A_ current amplitude. ***D***, Plots of the mean current density of the *I*_A_ (peak *I*_A_/capacitance) vs the test step potential, showing no effect of Dex on the *I*_A_ current density. ***E***, Plots of the normalized chord conductance of the *I*_A_ vs the test step potential, showing no effect of Dex on the voltage dependence of activation of the *I*_A_. ***F***, Plots of the mean *I*_A_ 10–90% rise time vs test step potential; the Dex effect on the activation rate of *I*_A_ was not significant. ***G***, Representative traces showing voltage-dependent inactivation of *I*_A_ in response to voltage steps to −15 mV from 200 ms conditioning steps between −135 and −25 mV before and after 10 min of Dex application (1 μm). Dexamethasone had no effect on the inactivation voltage dependence of the *I*_A_. Inset, Inactivation voltage-clamp protocol. ***H***, Plots of the mean normalized current amplitude vs the conditioning step potential, showing no effect of DEX on the voltage dependence of *I*_A_ inactivation. All recordings were in PVN magnocellular neurons in slices from untreated rats.

We used two separate voltage protocols to isolate the *I*_A_ in PVN magnocellular neurons (Fig. 1*A*). The first protocol included a 200 ms hyperpolarizing conditioning step to −115 mV, which removed the inactivation of the *I*_A_, followed by sequential 200 ms depolarizing test pulses to between −75 and +35 mV in 10 mV increments, which resulted in the activation of both the *I*_A_ and high voltage-activated potassium current (*I*_K_). The second protocol consisted of the same series of test pulses, but from a 200 ms depolarizing conditioning step to −45 mV. The *I*_K_ was isolated from the *I*_A_ in the second protocol because the *I*_A_ in PVN magnocellular neurons is inactivated at −45 mV (Luther and Tasker, 2000). The *I*_A_ was isolated by digitally subtracting the current responses generated by the second protocol from those generated by the first protocol, which removed the *I*_K_, leak current, and capacitive currents. The inactivation of the *I*_A_ was then studied with a third voltage protocol, consisting of sequential 200 ms conditioning steps to between −135 and −25 mV in 10 mV increments, which removed a variable amount of inactivation of *I*_A,_ followed by a command step to −15 mV. We introduced a small step, to −75 mV for 5 ms, between the conditioning steps and the test steps in both protocols, which allowed all test steps to be delivered from the same initial voltage level. This way the voltage-clamp artifact was the same size and pulses were all more conveniently subtracted from each other or compared. We tested the effect of this short pretest step on *I*_A_ and did not observe it to change *I*_A_ significantly, since very little *I*_A_ was activated at −75 and 5 ms was not sufficient to inactivate *I*_A_ (Luther and Tasker, 2000).

### Drug application

Water-soluble forms of the steroids dexamethasone (Dex; dexamethasone−cyclodextrin complex, 1 μm) and corticosterone (corticosterone, 2-hydroxypropyl-β-cyclodextrin, 1 μm; Sigma-Aldrich) were directly dissolved in aCSF to their final concentrations and applied in the bath perfusion. The Dex−bovine serum albumin (BSA) conjugate (10 μm) was dissolved in aCSF with 25% β-cyclodextrin (Sigma-Aldrich) as a carrier to increase its solubility. The concentration of Dex−BSA (10 μm) was selected to obtain an effective concentration of Dex of ∼1 μm, as the BSA conjugate had a steroid-to-BSA ratio of 8:1. TTX (1 μm; Sigma-Aldrich) and CdCl_2_ (200 µm; Sigma-Aldrich) were dissolved in sterile water and stored in 10 mm stock solutions at −20°C, and were dissolved to their final concentrations in aCSF immediately before bath application. The endogenous cannabinoids anandamide (AEA) and 2-arachidonoylglycerol (2-AG; Tocris Bioscience) were dissolved in DMSO, stored in 10 mm stock solutions at −20°C, and dissolved to their final concentrations in aCSF just before their application in the bath. The cannabinoid receptor inverse agonists *N*-(piperidin-1-yl)-5-(4-iodophenyl)-1-(2,4-dichlorophenyl)-4-methyl-1H-pyrazole-3-carboxamide (am251; 1 μm; Tocris Bioscience) and rimonobant (SR141716, 1 μm; provided by the National Institute of Mental Health Chemical Synthesis and Drug Supply Program) were stored as 10 mm stock solutions in DMSO at −20°C and dissolved to their final concentrations in aCSF before bath application. The vanilloid receptor agonist capsaicin and the antagonist capsazepine (Tocris Bioscience) were stored as 10 mm stock solutions in DMSO at −20°C and dissolved to their final concentrations in aCSF before bath application. The protein synthesis inhibitor cycloheximide (CHX; Tocris Bioscience) was dissolved in sterile water and stored in a 10 mm stock solution at −20°C. Fluorocitrate (D,L-fluorocitric acid, barium salt; 100 µm; Sigma-Aldrich) was dissolved in aCSF with 15 min of sonication. The DMSO and β-cyclodextrin vehicles without the cannabinoids or glucocorticoids had no effect on the wave form of the *I*_A_.

### Statistical methods and curve fitting

Values are expressed as the mean ± SEM. Statistical analyses were performed using a two-way ANOVA with the Bonferroni multiple comparisons test for between-group comparisons and the Student’s paired *t* test for within-cell comparisons. Differences were considered significant at *p* < 0.05. Boltzmann and exponential fits were used to fit data plots and current traces using the fitting methods provided in the GraphPad Prism software (GraphPad Software) and Clampfit 9 (pCLAMP 9, Molecular Devices), respectively.

## Results

### Rapid glucocorticoid modulation of *I*_A_

Corticosteroids rapidly modulate postsynaptic voltage-gated potassium currents in hippocampal pyramidal neurons (Olijslagers et al., 2008). Magnocellular neuroendocrine cells of the hypothalamic PVN generate a prominent *I*_A_ that shapes their pattern of action potential firing (Luther and Tasker, 2000; Ellis et al., 2007). We first tested for a rapid effect of glucocorticoids on the activation and inactivation of the *I*_A_ in magnocellular neurons. The passive properties of the recorded magnocellular neurons are presented in Table 1. The synthetic glucocorticoid Dex, applied at a concentration determined in previous studies to be near saturating for rapid actions on synaptic transmission (1 μm; Di et al., 2003, 2005a), had no effect on the *I*_A_. Based on a two-way ANOVA (*p* > 0.05, *n* = 9), there was no change at the end of a 10 min bath application of Dex in the *I*_A_ mean current amplitude, the *I*_A_ current density, or the voltage dependence of *I*_A_ activation (Fig. 1*B–E*). Dex also had no effect on the activation kinetics of the *I*_A_ (10–90% rise time measured with voltage steps from −115 to −25 mV; *p* > 0.05, Student’s paired *t* test; Fig. 1*F*) or on the voltage dependence of *I*_A_ inactivation (*p* > 0.05, two-way ANOVA; Fig. 1*G*,*H*). The rate of inactivation was examined by fitting the *I*_A_ decay phase with a single exponential function to determine the inactivation time constant, which was also unchanged by Dex treatment (data not shown; *p* > 0.05, Student’s paired *t* test). Dex had no effect on the amplitude, current density, or voltage dependence of the activation of the *I*_K_ (*p* > 0.05, ANOVA; data not shown).

**Table 1.** Effects of chronic dehydration on passive electrical properties of magnocellular neurons

	Untreated (*n* = 9)	Dehydrated (*n* = 15)
Membrane capacitance (pF)	24.3 ± 2.5	40.0 ± 4.4*
Membrane resistance (MΩ)	794.3 ± 48.4	667.5 ± 51.0*
Holding current (pA)	17.7 ± 3.8	13.4 ± 5.0*

* *p* > 0.05 vs neurons from untreated rats, Student’s unpaired *t* test.

**Figure 2.**
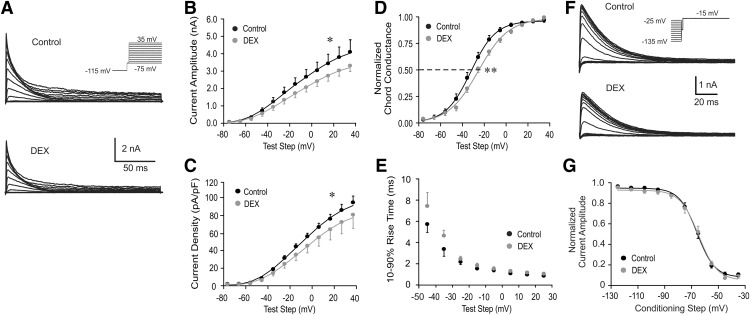
Rapid glucocorticoid modulation of *I*_A_ in PVN magnocellular neurons from salt-loaded rats. ***A***, Representative traces showing voltage-dependent activation of *I*_A_ in PVN magnocellular neurons from salt-loaded rats before and at the end of a 10 min Dex application (1 µm). Inset, Activation voltage-clamp protocol. ***B***, Plots of the mean peak amplitude of the *I*_A_ vs the test step potential; Dex caused a significant reduction in the *I*_A_ current amplitude. ***C***, Plots of the mean current density (peak current/capacitance) of the *I*_A_ vs the test step potential; Dex caused a significant reduction in the *I*_A_ current density. ***D***, Plots of the normalized chord conductance of the *I*_A_ vs the test step potential; Dex caused a significant shift to the right in the voltage dependence of activation of the *I*_A_. ***E***, Plots of the mean 10–90% *I*_A_ rise time vs the test step potential; Dex did not change the *I*_A_ activation kinetics. ***F***, Representative traces showing voltage-dependent inactivation of *I*_A_ in response to voltage steps to −15 mV from 200 ms conditioning steps between −135 and −25 mV before and at the end of a 10 min Dex application (1 μm). Inset, Inactivation voltage-clamp protocol. ***G***, Plots of the mean normalized current amplitude vs the conditioning potential. Dexamethasone had no effect on the voltage dependence of *I*_A_ inactivation. All recordings were in PVN magnocellular neurons in slices from salt-loaded rats. *p < 0.05; **p < 0.01 with ANOVA.

Chronic dehydration causes loss of glial coverage; enhanced glutamatergic, GABAergic, and noradrenergic synaptic regulation of magnocellular neurons; and altered voltage-gated currents (Tasker et al., 2017). We next tested the possibility that glucocorticoid modulation of the *I*_A_ in PVN magnocellular neurons is altered under conditions of chronic dehydration caused by salt loading. Five to 7 days of salt loading caused an increase in the membrane capacitance and a decrease in the input resistance of magnocellular neurons (Table 1), which are characteristic of dehydration-induced hypertrophy in the magnocellular neurons (Miyata and Hatton, 2002; Di and Tasker, 2004; Shah et al., 2014). Unlike neurons from untreated rats, bath application of Dex (1 μm) to PVN magnocellular neurons (*n* = 15) in slices from salt-loaded rats caused decreases in both the *I*_A_ current amplitude and the *I*_A_ current density (*p* < 0.05, two-way ANOVA followed by the Bonferroni multiple comparisons test for each test step; Fig. 2*A–C*) and shifted the activation curve of *I*_A_ positive by 4.9 mV (*p* < 0.01; Student’s paired *t* test; Fig. 2*D*) within 10 min. The *I*_A_ 10–90% rise time (Fig. 2*E*) and the voltage dependence of *I*_A_ inactivation (Fig. 2*F*,*G*) were unchanged. Like magnocellular neurons from untreated rats, Dex had no effect on the amplitude, current density, or voltage dependence of activation of the *I*_K_ in magnocellular neurons recorded in slices from salt-loaded rats (data not shown).

**Figure 3.**
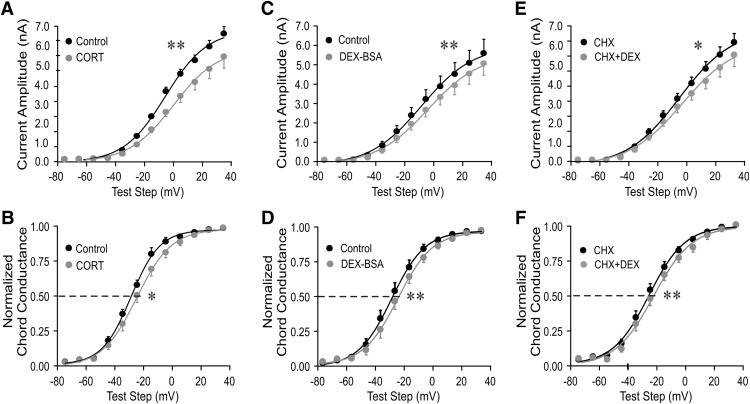
The rapid glucocorticoid effect on *I*_A_ in magnocellular neurons from salt-loaded rats was mediated by a membrane-associated receptor. ***A–F***, Plots of the mean current amplitude of the *I*_A_ (***A***, ***C***, ***E***) and the *I*_A_ voltage dependence of activation (normalized chord conductance; ***B***, ***D***, ***F***) vs the test step potential before and at the end of a 10 min application of the endogenous glucocorticoid corticosterone (Cort), the membrane-impermeant glucocorticoid Dex–BSA, and Dex in the presence of the protein translation inhibitor CHX. The endogenous glucocorticoid Cort (***A***, ***B***), and the membrane-impermeant glucocorticoid Dex–BSA (***C***, ***D***) reduced the *I*_A_ peak amplitude and shifted the *I*_A_ voltage dependence to the right. Blocking protein synthesis (***E***, ***F***) failed to block the glucocorticoid modulation of the *I*_A_. *p < 0.05; **p < 0.01 with ANOVA in ***A***, ***C***, ***E*** and with Student’s paired *t* test in ***B***, ***D***, ***F***.

The endogenous glucocorticoid corticosterone had a similar effect on the *I*_A_ activation in MNCs from dehydrated rats. A 10 min bath application of corticosterone (1 μm) reduced the *I*_A_ mean current amplitude (*p* < 0.01, two-way ANOVA, Bonferroni’s test; Fig. 3*A*) and induced a 4.3 mV positive shift in the *I*_A_ half-activation potential in MNCs from dehydrated rats (*n* = 10; *p* < 0.05, Student’s paired *t* test; Fig. 3*B*). Corticosterone had no effect on the voltage dependence of inactivation or on the inactivation time constant of *I*_A_ (data not shown).

Rapid glucocorticoid modulation of synaptic inputs to hypothalamic magnocellular and parvocellular neuroendocrine cells are mediated by a membrane-associated receptor (Di et al., 2003, 2005a). Here, we investigated whether the rapid glucocorticoid modulation of the *I*_A_ in PVN magnocellular neurons from dehydrated rats is also mediated by a membrane-associated glucocorticoid receptor. Bath application of the membrane-impermeant Dex–BSA conjugate (10 μm), like Dex, reduced the mean current amplitude (*p* < 0.01, two-way ANOVA followed by Bonferroni multiple comparisons test for each test step, *n* = 7; Fig. 3*C*) and caused a 3.4 mV positive shift in the *I*_A_ half-activation potential (*n* = 7; *p* < 0.01, Student’s paired *t* test; Fig. 3*D*). This implicated a membrane site of steroid action. We used a 10-fold higher concentration of Dex–BSA because the Dex-to-BSA ratio of the Dex–BSA compound was 8:1, and we assumed that a single Dex molecule per Dex–BSA was available to bind to the receptor. The weaker effect of the Dex–BSA compared with Dex could be due to a lower effective concentration of available Dex to bind to the receptor or to a steric hindrance of Dex binding by the Dex–BSA three-dimensional structure. We have shown recently that free molecules of Dex do not dissociate from the Dex–BSA conjugate to penetrate into the cell (Weiss et al., in press).

To determine whether the rapid glucocorticoid effect on *I*_A_ in MNCs from dehydrated rats is independent of *de novo* protein synthesis, we tested the sensitivity of the Dex effect to the protein synthesis inhibitor cycloheximide. Following a 30 min preincubation of slices in cycloheximide (10 μM), the Dex-induced suppression of *I*_A_ amplitude (*p* < 0.05, two-way ANOVA, Bonferroni’s *post hoc* test; *n* = 7; Fig. 3*E*) and positive shift in *I*_A_ half-activation potential (by 3.2 mV; *p* < 0.01, Student’s paired *t* test; *n* = 7; Fig. 3*F*) within 10 min were maintained. This indicated that the Dex effect on *I*_A_ does not require protein synthesis and is mediated by a nontranscriptional mechanism.

### Endocannabinoid dependence of the glucocorticoid modulation of the *I*_A_

**Figure 4.**
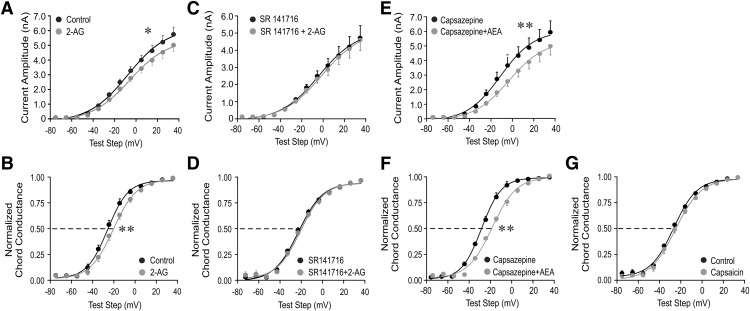
CB1 receptor agonists mimicked the glucocorticoid modulation of *I*_A_. ***A***, ***B***, Bath application of the endocannabinoid 2-AG (1 μm) elicited a significant decrease in the *I*_A_ mean peak amplitude (***A***) and a positive shift in the *I*_A_ activation voltage dependence (normalized chord conductance; ***B***) in magnocellular neurons from untreated rats. ***C***, ***D***, The CB1 receptor inverse agonist SR141716 (rimonabant, 1 μm) blocked the decrease in mean *I*_A_ peak amplitude (***C***) and the rightward shift in the chord conductance curve (***D***). ***E***, ***F***, AEA elicited a decrease in the *I*_A_ mean peak amplitude (***E***) and a positive shift in the *I*_A_ activation voltage dependence (chord conductance; ***F***) in the presence of the TRPV1 receptor antagonist capsazepine (1 μm). ***G***, The TRPV1 agonist capsaicin (1 μm) had no effect on the *I*_A_ voltage dependence of activation (chord conductance). *p < 0.05; **p < 0.01 with ANOVA in ***A***, ***C***, ***E*** and with Student’s paired *t* test in ***B***, ***D***, ***F***, ***G***.

Rapid glucocorticoid modulation of glutamatergic synaptic inputs to magnocellular and parvocellular neuroendocrine cells of the hypothalamus are mediated by the retrograde release of an endocannabinoid (Di et al., 2003, 2005a). Here, we tested for the endocannabinoid dependence of the rapid glucocorticoid modulation of the postsynaptic *I*_A_ in PVN magnocellular neuroendocrine cells. Bath application of the endocannabinoid 2-AG (1 μm) caused a decrease in the mean *I*_A_ peak amplitude (*p* < 0.05, two-way ANOVA, Bonferroni multiple comparisons test; *n* = 16; Fig. 4*A*) and a 5 mV positive shift in the *I*_A_ half-activation potential (*p* < 0.01, Student’s paired *t* test; *n* = 16; Fig. 4*B*) in PVN magnocellular neurons from untreated rats. This effect of 2-AG on the *I*_A_ was blocked by the CB1 receptor inverse agonist SR141716 (1 μm; *n* = 6; Fig. 4*C*,*D*).

The other main endocannabinoid, AEA, activates both CB1 receptors and transient receptor potential-vanilloid 1 (TRPV1) receptors (Marinelli et al., 2003; Derbenev et al., 2006), so we blocked TRPV1 receptors with the TRPV1 receptor antagonist capsazepine during AEA application to test AEA modulation of *I*_A_ via activation of the CB1 receptor. Preapplication of capsazepine (1 μm) 10 min before AEA application had no effect on the *I*_A_ in magnocellular neurons. AEA (1 μm) added to the capsazepine in the bath solution caused a decrease in the *I*_A_ peak amplitude (*p* < 0.01, two-way ANOVA, Bonferroni multiple-comparisons test; *n* = 7; Fig. 4*E*) and induced a 10 mV positive shift in the *I*_A_ half-activation potential (*p* < 0.01, Student’s paired *t* test; *n* = 7; Fig. 4*F*). The TRPV1 agonist capsaicin (1 μm) had no effect on the voltage dependence of *I*_A_ activation (Fig. 4*G*). These data indicate that CB1 receptor activation with endocannabinoids modulates *I*_A_ activation in a manner similar to glucocorticoids.

Unlike glucocorticoids, which had no effect on the *I*_A_ in magnocellular neurons from normally hydrated rats, exogenously applied endocannabinoids induced similar effects on the *I*_A_ in magnocellular neurons from both normally hydrated and salt-loaded rats. Similar to magnocellular neurons from untreated rats, exogenous application of 2-AG (1 μm) to magnocellular neurons from salt-loaded rats caused a significant decrease in the mean peak current amplitude (*p* < 0.05, two-way ANOVA, Bonferroni multiple comparisons test) and shifted the activation curve of *I*_A_ to the right by 3.9 mV (*p* < 0.05, Student’s paired *t* test, compared with 5 mV in untreated rats). Exogenous application of the other main endocannabinoid, AEA (0.5 μm), also decreased the *I*_A_ peak amplitude (*p* < 0.05, two-way ANOVA, Bonferroni multiple comparisons test; *n* = 11), and shifted the *I*_A_ half-activation potential positive by 4.1 mV (*p* < 0.01, Student’s paired *t* test; *n* = 11) in PVN magnocellular neurons from salt-loaded rats.

**Figure 5.**
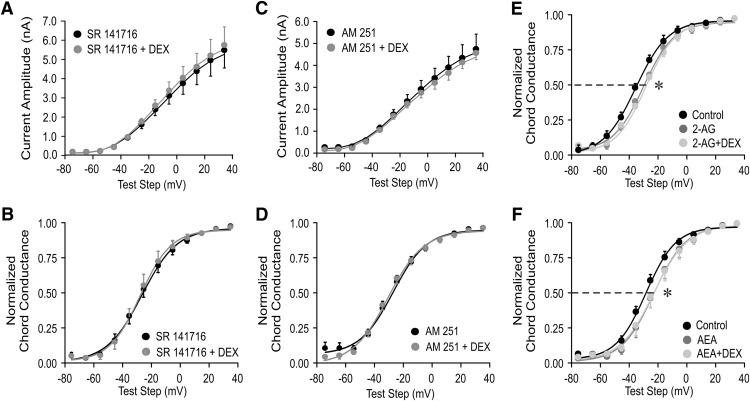
CB1 receptor analogs blocked the glucocorticoid modulation of *I*_A_. ***A***, ***B***, The CB1 receptor inverse agonist SR141716 (1 μm) blocked the decrease in *I*_A_ peak amplitude (***A***) and the rightward shift in the voltage dependence of activation (normalized chord conductance; ***B***) induced by Dex (1 μm) in PVN magnocellular neurons in slices from salt-loaded rats. ***C***, ***D***, The CB1 receptor inverse agonist am251 also blocked the Dex-induced decrease in *I*_A_ peak amplitude (***C***) and the positive shift in activation voltage dependence (normalized chord conductance; ***D***) in PVN magnocellular neurons from salt-loaded rats. ***E***, ***F***, 2-AG (1 μm; ***E***) and AEA (1 μm; ***F***) caused a positive shift in the *I*_A_ voltage-dependence curve (normalized chord conductance) from the control value, but subsequent application of Dex (1 μm) failed to further shift the curve, suggesting occlusion of the Dex effect by prior CB1 receptor activation. *p < 0.05 with ANOVA. Student’s paired *t* test.

We next tested whether the postsynaptic modulation of the *I*_A_ by glucocorticoids in magnocellular neuroendocrine cells from salt-loaded rats is dependent on CB1 receptor activation. The CB1 receptor inverse agonists am251 (1 µm) and SR141716 (1 µm) by themselves had no effect on *I*_A_ amplitude or activation voltage dependence (data not shown), but both am251 and SR141716 blocked the Dex-induced decrease in *I*_A_ peak current amplitude (Fig. 5*A*,*C*) and the rightward shift in the *I*_A_ activation curve (Fig. 5*B*,*D*) in MNCs in slices from salt-loaded rats, suggesting the involvement of endocannabinoid in the rapid glucocorticoid modulation of *I*_A_.

We then tested whether glucocorticoids and endocannabinoids act on the *I*_A_ through the same signaling pathway or through separate, parallel pathways by testing for occlusion of the glucocorticoid-induced modulation of *I*_A_ by previous endocannabinoid exposure. In slices from salt-loaded rats, 2-AG applied alone caused a decrease in the *I*_A_ peak current amplitude (*p* < 0.05, two-way ANOVA, Bonferroni multiple comparisons test; *n* = 4) and a 5.5 mV positive shift in the *I*_A_ half-activation potential (*p* < 0.05, Student’s paired *t* test; *n* = 4). The subsequent application of Dex (1 μm), in the presence of 2-AG (1 μm), failed to further decrease the peak *I*_A_ amplitude (*p* > 0.05, ANOVA) or to further shift the *I*_A_ half-activation potential to the right (*p* > 0.05, Student’s paired *t* test; *n* = 4; Fig. 5*E*). Similarly, AEA decreased the *I*_A_ peak current amplitude (*p* < 0.05, two-way ANOVA, Bonferroni multiple comparisons test; *n* = 4) and shifted the *I*_A_ half-activation potential positive by 5 mV (*p* < 0.05, Student’s paired *t* test; *n* = 4). Dex (1 μm) failed to decrease the peak current amplitude further (*p* > 0.05, ANOVA) or elicit any further rightward shift in the *I*_A_ half-activation potential in the presence of AEA (*p* > 0.05, Student’s paired *t* test; *n* = 4; Fig. 5*F*).

### Possible glial regulation of the glucocorticoid modulation of *I*_A_

**Figure 6.**
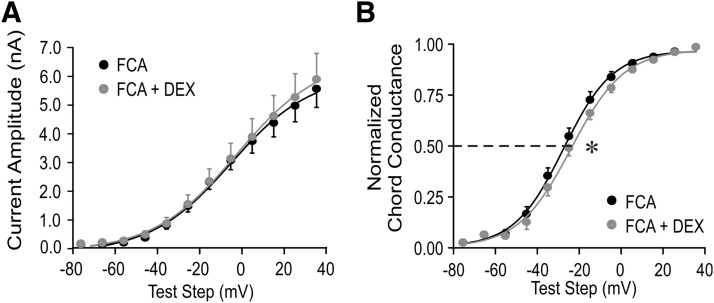
Glucocorticoid modulation of *I*_A_ following gliotoxin treatment. Brain slices from untreated rats were preincubated in the gliotoxin fluorocitrate for ∼2 h before recordings. ***A***, Following fluorocitrate treatment, Dex had no effect on the *I*_A_ mean peak amplitude. ***B***, Dex caused a positive shift in the voltage dependence of *I*_A_ activation (normalized chord conductance) in fluorocitrate-treated slices. *p < 0.05, ANOVA. Student’s paired *t* test.

Chronic dehydration causes a structural change in astrocytic morphology that results in a reduction in the coverage of neurons and synapses by astrocytic processes (Miyata et al., 1994; Theodosis et al., 1995; Hatton, 1997; Tasker et al., 2017) and leads to spillover of synaptic signals to extrasynaptic sites (Boudaba et al., 2003; Fleming et al., 2011). We have found that salt loading causes spillover of endocannabinoid from excitatory synapses to inhibitory synapses, which was mimicked by suppressing glial activity with a gliotoxin (Di et al., 2013). The emergence of rapid glucocorticoid modulation of the *I*_A_ with dehydration suggests a possible role for glial coverage in the control of autocrine actions of endocannabinoids. To test for a role of astrocyte buffering in the glucocorticoid–endocannabinoid modulation of *I*_A_ channels, we used the gliotoxin fluorocitrate to impair astrocyte buffering capability. Fluorocitrate is preferentially taken up by glia and blocks glial metabolic activity, including membrane transport, by inhibiting the citric acid cycle (Clarke, 1991; Gordon et al., 2005). Slices from normally hydrated rats were preincubated for ∼2 h in fluorocitrate (100 µm). Dexamethasone (1 μm) had a small but significant effect on the *I*_A_ in PVN magnocellular neurons in slices from untreated rats exposed to fluorocitrate. Whereas Dex had no effect on the *I*_A_ peak current amplitude following fluorocitrate treatment (*p* > 0.05, ANOVA; *n* = 8; Fig. 6*A*), it caused a small, but significant, shift to the right in the *I*_A_ half-activation potential, by 3.2 mV (*p* < 0.05, Student’s paired *t* test; *n* = 8; Fig. 6*B*). Thus, blocking glial metabolism in slices from untreated rats partially reproduced the effect of *in vivo* salt loading on the glucocorticoid modulation of the *I*_A_.

## Discussion

Glucocorticoids rapidly stimulate the synthesis and dendritic release of endocannabinoids, which act retrogradely to modulate glutamate release from excitatory synaptic terminals onto PVN neuroendocrine cells (Di et al., 2005b, 2009). In addition to their well characterized presynaptic actions, cannabinoids have also been shown to modulate postsynaptic potassium currents (Deadwyler et al., 1995; Tang et al., 2005), although, to our knowledge, the regulation of postsynaptic conductance by endogenously released cannabinoids has not been reported. Here, we demonstrate a glucocorticoid-induced suppression of the A-type potassium current in hypothalamic magnocellular neuroendocrine cells that is CB1 receptor dependent, suggesting a postsynaptic autocrine action of endogenously released cannabinoid. The glucocorticoid modulation of *I*_A_ occurred rapidly, within minutes of its introduction, and reached saturation within 10 min. The glucocorticoid effect was maintained with the membrane-impermeant Dex–BSA conjugate, indicating that it was mediated by a membrane-associated receptor, and it was not blocked by inhibiting protein synthesis, suggesting a nongenomic mechanism. Therefore, like the rapid glucocorticoid effects on glutamate and GABA neurotransmission in the PVN (Di et al., 2003, 2009), these findings implicate a membrane glucocorticoid receptor in the postsynaptic modulation of *I*_A_.

We have performed concentration–response analysis on the rapid Dex-induced endocannabinoid production and retrograde suppression of excitatory synaptic transmission in magnocellular neurons (Di et al., 2005a). In that study, we found that the glucocorticoid-induced endocannabinoid synthesis in magnocellular neurons has a half-effective concentration of ∼350 nm and a saturating concentration near 1 μm. We used the near-saturating concentration of 1 μm for Dex in this study to approximate the high circulating glucocorticoid levels encountered following stress activation of the hypothalamic–pituitary–adrenal axis. The 1 μm concentration of Dex and corticosterone that we used is approximately equivalent to 350–400 ng/ml concentration of circulating glucocorticoid, which is within physiologic limits. This concentration was ineffective in the magnocellular neurons from untreated rats, which suggested that the effect seen in the magnocellular neurons from salt-loaded rats was not the result of a nonspecific action of the steroid.

We showed previously that glucocorticoids stimulate the synthesis of endocannabinoids in the PVN (Malcher-Lopes et al., 2006) and that they suppress excitatory synaptic inputs to PVN and SON neurons via the dendritic release and retrograde actions of endocannabinoids (Di et al., 2003, 2005a). Here we demonstrate the endocannabinoid dependence of the glucocorticoid modulation of the *I*_A_, which suggests that the glucocorticoid-induced release of endocannabinoid also regulates postsynaptic conductance in these cells and is consistent with cannabinoid modulation of *I*_A_ in other brain areas (Deadwyler et al., 1995; Tang et al., 2005). In addition to their retrograde actions, therefore, glucocorticoid-induced endocannabinoids are capable of acting in either an autocrine or a paracrine fashion to regulate postsynaptic potassium currents in the PVN.

One possible mechanism of the negative modulation of the *I*_A_ by endocannabinoids is that the activation of postsynaptic CB1 receptors triggers a change in the phosphorylation state of the A-type potassium channels. There is a large body of evidence that supports the modulation of *I*_A_ by phosphorylation (Covarrubias et al., 1994; Birnbaum et al., 2004; Cai et al., 2005), and CB1 receptors are known to negatively couple via G_i_ to adenylyl cyclase activity, cAMP production, and PKA-dependent protein phosphorylation (Howlett et al., 2004). A decrease in the PKA-dependent phosphorylation of K_v_ channel subunits may result in a decrease in the ability of the voltage sensor of the *I*_A_ channel to respond to changes in membrane voltage (Park et al., 2006; Yang et al., 2007). Another possibility is that CB1 receptor activation leads to the modulation of one or more of the K_v_ channel auxiliary proteins, which are well known to regulate the *I*_A_ (Covarrubias et al., 1994; Schrader et al., 2009).

The rapid glucocorticoid modulation of the *I*_A_ was seen only in magnocellular neurons from rats subjected to chronic salt loading, a treatment known to induce the retraction of astrocytic processes from around magnocellular neurons (Theodosis et al., 1995; Tasker et al., 2017) and result in the synaptic spillover of endocannabinoid from excitatory to inhibitory synapses (Di et al., 2013). The emergent sensitivity to rapid glucocorticoid-induced endocannabinoid modulation of the *I*_A_ could be caused either by dehydration-induced plasticity of postsynaptic endocannabinoid or glucocorticoid signaling mechanisms or by the loss of glial endocannabinoid buffering due to astrocytic retraction. The neuronal–glial structural plasticity in magnocellular neurons under stimulated conditions (Theodosis et al., 1986; Miyata et al., 1994; Hatton, 1997) is accompanied by increasing levels of ambient neurotransmitters, such as glutamate (Oliet et al., 2001; Boudaba et al., 2003) and increasing heterosynaptic and extrasynaptic spillover of neurotransmitters (Piet et al., 2004). We have found that the glutamate synapse-specific effects of glucocorticoid-induced retrograde endocannabinoid actions (Di et al., 2009) are controlled by glial coverage and that, following dehydration-induced glial retraction, endocannabinoid also accesses and activates CB1 receptors on GABA synapses (Di et al., 2013). Our findings here are consistent with glucocorticoid-induced autocrine endocannabinoid actions that are constrained by glial restriction of extracellular endocannabinoid diffusion. That the *I*_A_ modulation by exogenously applied cannabinoids was similar in slices from both untreated and salt-loaded rats suggests that the lack of effect of endogenously released endocannabinoid in slices from untreated rats was not due to a change in the sensitivity of endocannabinoid signaling with dehydration. While these findings do not exclude the possibility of a change in the sensitivity to rapid glucocorticoid signaling with salt loading, they may be explained by dehydration-induced glial retraction that expands the extracellular diffusional reach of the endocannabinoid to allow access to postsynaptic CB1 receptors that are normally inaccessible due to astrocytic coverage. However, a role for astrocytes in limiting the glucocorticoid-induced autocrine endocannabinoid actions is still speculative, as it was only partially supported by our recordings in slices in which glial metabolism, and thus endocannabinoid reuptake, was inhibited by the gliotoxin fluorocitrate (Clarke, 1991; Gordon et al., 2005). Thus, fluorocitrate treatment partially recapitulated the dehydration-induced facilitation of glucocorticoid modulation of the *I*_A_, as Dex shifted the *I*_A_ activation curve but did not suppress the peak *I*_A_ following fluorocitrate preincubation. Therefore, additional experiments are required to conclusively rule out possible plastic changes in glucocorticoid or endocannabinoid signaling in favor of the loss of astrocytic buffering to explain the emergence of rapid glucocorticoid modulation of the *I*_A_ with salt loading.

In magnocellular neurons from chronically dehydrated rats, we observed a glucocorticoid-induced decrease in the peak amplitude and rightward shift in the voltage dependence of activation of the *I*_A_. The rightward shift in the activation curve of the *I*_A_, with no change in the inactivation voltage dependence, means that a greater depolarizing stimulus is required to activate the transient potassium current and that fewer A-type potassium channels are activated with depolarization in the range of the action potential threshold. The lower peak amplitude of the *I*_A_ indicates that significantly less current will be activated with depolarization. The glucocorticoid modulation of the *I*_A_, therefore, should increase the excitability of magnocellular neurons following chronic dehydration. Contrary to our expectations, Dex had no effect on depolarization-induced spiking in neurons from salt-loaded rats. This may be because dehydration itself causes a reduction in the *I*_A_ current density (N. Wu and J. G. Tasker, unpublished observation), as well as changes in other voltage-gated conductances (Tanaka et al., 1999; Zhang et al., 2007), which makes it difficult to isolate the impact of glucocorticoid modulation of *I*_A_ on firing properties.

Glucocorticoid suppression of the *I*_A_ in magnocellular neurons during chronic dehydration should enhance the effect of synaptic excitation. Glucocorticoid-induced retrograde endocannabinoid release suppresses presynaptic glutamate release and nitric oxide release enhances presynaptic GABA release onto magnocellular neurons from normally hydrated rats (Di et al., 2005a, 2009). This should negate any excitatory effect of suppressing the *I*_A_; however, the glucocorticoid facilitation of GABA release is lost during dehydration due to the spillover inhibitory actions of endocannabinoid at GABA synapses (Di et al., 2013). Therefore, the excitation–inhibition balance established by glutamate and GABA release and *I*_A_ activation in the dendrites would be tilted toward excitation. This combined with the increased sensitivity to noradrenergic inputs (Di and Tasker, 2004) should enhance or maintain magnocellular neuron activity and neurohypophysial hormone release under conditions of osmotic stress (Hatton, 1997). Collectively, the different effects of glucocorticoids on magnocellular neurons in untreated and chronically dehydrated rats reveal a mechanism for the neuroendocrine system to respond to stress and maintain homeostasis by means of integration of the afferent neural circuit information and circulating hormonal signals.

## Synthesis

Reviewing Editor: Jeffrey Blaustein, University of Massachusetts Decisions are customarily a result of the Reviewing Editor and the peer reviewers coming together and discussing their recommendations until a consensus is reached. When revisions are invited, a fact-based synthesis statement explaining their decision and outlining what is needed to prepare a revision will be listed below. The following reviewer(s) agreed to reveal their identity: Scott Thompson.

Although there were a number of issues, the editor and reviewers agreed that the experiments were well done. With proper revision, this paper could be acceptable. The biggest weakness was considered to be that “A change in glial uptake in endocannabinoids is implicated as the cause, although the results are equivocal and other alternative hypotheses are not considered. While the experiments have been carefully performed and reported, the context and conclusions of the paper are less than fully satisfying.”

Reviewer 1:

1) Significance Statement, 6th line- should read “via a novel....” “Not vis a model”. And the last sentence should be changed to read “changes.......lead.....”, not “leads”.

2) Results- Figure 3- there is no text in legend for G.

3) Results- Figures 3-6. Why are the histograms in these figures in % change- rather than the actual values measured? What is the control to which the % change is compared? The significance (*) seems to refer to differences from these groups to controls as suggested in the Results. Shouldn't the authors compare these different % change values between (or among) the groups, to test whether that % change is statistically different?

4) Results- under Glial regulation- first line should read “that results” not “that is results”.

5) Discussion- The authors state on page 19 that “Contrary to our expectations, Dex had no effect on spiking in neurons from salt-loaded rats”. But no data are presented in the ms on spiking. Furthermore, since the data on Ia for controls and salt-loaded animals are actually contained in the experiments reported herein, the authors should statistically compare Ia in control and stressed rats, rather than speaking about “unpublished observations” showing a reduction in Ia during dehydration.

6) Discussion- The statements on page 20 about the dendritic localization of Ia and these effects should be more clear. The authors are mixing localization of the current, with localization of the effects of steroids and CBs. Since magnocellular dendrites account for the great majority of neuronal surface area, the reduction in Ia in dissociated cells to only 1 third of original is hardly surprising, and is not in itself evidence for any selective distribution of Ia in dendrites. Also citing the Widmer et al (1997) paper here is specious. This paper suggested that Ia was localized in vasopressin neuron dendrites, but was clearly present on the oxytocin somata recorded in that paper. But even this result was not confirmed- a later paper clearly showed Ia in both vasopressin and oxytocin dissociated neurons, and it was actually much greater in vasopressin neurons, independent of cell size (Fisher et al., 1998). Thus the unpublished data mentioned here should only speak to the localization of effects of CBs and Dex, not to the localization of Ia per se. In this regard, it is unfortunate that vasopressin and oxytocin neurons were not assessed separately. Should both populations be expected to react similarly in this regard?

Reviewer 2:

1. The only evidence supporting an involvement of glia is the use of a putative gliotoxin, FCA. It would be helpful to show that an inhibitor of endocannabinoid uptake or catabolism, such as a FAAH inhibitor also revealed endocannabinoid sensitivity in normally hydrated rats.

2. It is dissatisfying, as the authors themselves discuss, that the functional consequences of the effects so carefully described here are completely unclear. The authors point out that dehydration-induced changes in synaptic transmission should negate the effects of ECs on I-A. The downregulation of I-A has no effect on evoked spiking. The authors did not look at whether the observed I-A changes contribute to altered HPA axis function. This should be discussed in the manuscript.

3. The authors suggest that PKA-dependent mechanisms may underlie the EC effect, but fail to provide any evidence to support this readily testable hypothesis. Although this evidence would be desirable, it is not necessary for publication of this paper. Nevertheless, the issue should be addressed.

4. Chronic dehydration is a strong stressor and presumably affects on-going PVN activity. The authors should consider whether such changes in on-going PVN activity might also affect EC-IA coupling and address this in the manuscript.

5. The authors need to justify the use of 1uM corticosterone. This is, by my calculation, about 1000 times higher than peak stress-induced cort levels.

6. I do not understand the graphs in Figs 3G, 4E and 5E. How were the error bars computed? Why do the mean values (15-20 mV) seem so much greater than the shifts shown in the corresponding graphs (5-10mV)?

7. There is a typo in the figure legends: SR141716 is rimonobant not mifepristone

## Author Response

We thank the editor and reviewers for their careful reviews of our manuscript and for the constructive comments concerning how to improve it. We have highlighted all additions to the revised manuscript in red type face. As detailed below, we have made one significant subtraction in the text, of the discussion on dendritic localization of the A-type potassium channels, and have dropped the bar graphs depicting percent changes in half-activation voltage from Figures 3 through 6.

Synthesis Statement for Author

Although there were a number of issues, the editor and reviewers agreed that the experiments were well done. With proper revision, this paper could be acceptable. The biggest weakness was considered to be that “A change in glial uptake in endocannabinoids is implicated as the cause, although the results are equivocal and other alternative hypotheses are not considered. While the experiments have been carefully performed and reported, the context and conclusions of the paper are less than fully satisfying.”

We have attempted to downplay the possibility that glial uptake is responsible for the change in glucocorticoid-induced endocannabinoid modulation of the IA in dehydrated rat magnocellular neurons, and to provide possible alternative mechanisms, including plasticity in rapid glucocorticoid and/or endocannabinoid signaling.

Reviewer 1:

1) Significance Statement, 6th line- should read “via a novel....” “Not vis a model”. And the last sentence should be changed to read “changes.......lead.....”, not “leads”.

The errors have been corrected. Thank you for catching them.

2) Results- Figure 3- there is no text in legend for G.

Panel G in Figure 3 has been dropped (see below).

3) Results- Figures 3-6. Why are the histograms in these figures in % change- rather than the actual values measured? What is the control to which the % change is compared? The significance (*) seems to refer to differences from these groups to controls as suggested in the Results. Shouldn't the authors compare these different % change values between (or among) the groups, to test whether that % change is statistically different?

The histograms were meant to present the relative changes in half-activation voltage with respect to resting potential, but we agree that the measure was confusing, so we have dropped it in the figures. The voltage shift in the half-activation potential is reported in the text, so this was an additional way of expressing the shift that is not necessary.

4) Results- under Glial regulation- first line should read “that results” not “that is results”.

This has been corrected.

5) Discussion- The authors state on page 19 that “Contrary to our expectations, Dex had no effect on spiking in neurons from salt-loaded rats”. But no data are presented in the ms on spiking. Furthermore, since the data on Ia for controls and salt-loaded animals are actually contained in the experiments reported herein, the authors should statistically compare Ia in control and stressed rats, rather than speaking about “unpublished observations” showing a reduction in Ia during dehydration.

We did not present the current-clamp data showing spiking because Dex had no effect on the spiking properties of the magnocellular neurons. The changes in voltage-gated potassium currents caused by salt loading are the subject of a separate manuscript that is currently in revision, and which is significant in breadth. We respectfully argue that the comparison of IA properties in neurons from untreated and salt-loaded rats, which comprises a considerable amount of analysis, goes beyond the scope of this paper.

6) Discussion- The statements on page 20 about the dendritic localization of Ia and these effects should be more clear. The authors are mixing localization of the current, with localization of the effects of steroids and CBs. Since magnocellular dendrites account for the great majority of neuronal surface area, the reduction in Ia in dissociated cells to only 1 third of original is hardly surprising, and is not in itself evidence for any selective distribution of Ia in dendrites. Also citing the Widmer et al (1997) paper here is specious. This paper suggested that Ia was localized in vasopressin neuron dendrites, but was clearly present on the oxytocin somata recorded in that paper. But even this result was not confirmed- a later paper clearly showed Ia in both vasopressin and oxytocin dissociated neurons, and it was actually much greater in vasopressin neurons, independent of cell size (Fisher et al., 1998). Thus the unpublished data mentioned here should only speak to the localization of effects of CBs and Dex, not to the localization of Ia per se. In this regard, it is unfortunate that vasopressin and oxytocin neurons were not assessed separately. Should both populations be expected to react similarly in this regard?

We agree that this argument was speculative and a somewhat flimsy, so we have eliminated this discussion of the dendritic localization of the A-current channels and limited the discussion to the possible location of the glucocorticoid-induced endocannabinoid actions, as suggested. We have dropped the reference to our unpublished observations from recordings in dissociated cells, as these findings have not been fleshed out sufficiently to present here.

Reviewer 2:

1. The only evidence supporting an involvement of glia is the use of a putative gliotoxin, FCA. It would be helpful to show that an inhibitor of endocannabinoid uptake or catabolism, such as a FAAH inhibitor also revealed endocannabinoid sensitivity in normally hydrated rats.

We agree with the reviewer that the evidence supporting a role for glia in the glucocorticoid modulation if IA is inconclusive. In a parallel study, we performed experiments with FAAH and MAG lipase inhibitors and found that the pharmacology was problematic, so we prefer here to not go down that road and to focus rather on the emergence of autocrine actions of eCB, and to simply present the possibility that glia may be involved, with further experiments necessary to conclusively show this. We have changed the heading of this section of the Results to “Possible glial regulation of the glucocorticoid modulation of IA” and have added a sentence to the end of this section to reflect this. We have also tried to further emphasize in the Discussion that the role for glia in the mechanism is speculative and that further studies are required to determine this.

2. It is dissatisfying, as the authors themselves discuss, that the functional consequences of the effects so carefully described here are completely unclear. The authors point out that dehydration-induced changes in synaptic transmission should negate the effects of ECs on I-A. The downregulation of I-A has no effect on evoked spiking. The authors did not look at whether the observed I-A changes contribute to altered HPA axis function. This should be discussed in the manuscript.

Because we focused here on magnocellular neurons, we did not think to look at the effect of changes in IA on HPA activation in vivo. While it might be reasonable to expect chronic salt loading to alter HPA responses, it would be extremely challenging to attribute this effect to IA modulation in the magnocellular neurons. Since the changes in IA modulation in magnocellular neurons do not point to any obvious changes in HPA signaling, we respectfully suggest that it would be overly speculative at this point to discuss any possible effects on the HPA response.

3. The authors suggest that PKA-dependent mechanisms may underlie the EC effect, but fail to provide any evidence to support this readily testable hypothesis. Although this evidence would be desirable, it is not necessary for publication of this paper. Nevertheless, the issue should be addressed.

We agree that further dissection of the molecular mechanism of the endocannabinoid modulation of the IA would be interesting and would enhance the manuscript. Unfortunately, while the effect may be PKA-dependent, it may involve a more complicated signaling pathway, the modulation of different channel subunits, or the regulation of associated proteins. We prefer, therefore, to leave this to future, more in-depth study. We have discussed the possibility of cAMP-PKA modulation of the A-current channels and associated proteins.

4. Chronic dehydration is a strong stressor and presumably affects on-going PVN activity. The authors should consider whether such changes in on-going PVN activity might also affect EC-IA coupling and address this in the manuscript.

The reviewer is correct in assuming that chronic salt loading has strong effects on ongoing activity in the PVN. For example, we and others have found significant changes in the basal excitatory and inhibitory synaptic inputs to the magnocellular neurons, and a robust enhancement of the noradrenergic modulation of these inputs. In the revision, we have downplayed further the possible role of glial retraction and have tried to emphasize the fact that plastic changes in glucocorticoid and/or endocannabinoid signaling may be responsible for the emergent CB1 receptor-dependent modulation of the IA following chronic salt loading.

5. The authors need to justify the use of 1uM corticosterone. This is, by my calculation, about 1000 times higher than peak stress-induced cort levels.

We thank the reviewer for this suggestion, as this is an important consideration for interpretation of our findings, one that we neglected to discuss in the paper. We have now added a paragraph to the Discussion to explain the glucocorticoid concentration (1 uM) we used for our experiments in the context of physiological stress levels of glucocorticoid. By our calculations, 1uM corticosterone or dexamethasone corresponds approximately to stress levels of circulating glucocorticoid. At a molecular weight of 346.5 g/M, 1uM corticosterone is equivalent to approximately 346 ng/ml and 1-uM Dex (MW = 392.5 g/M) is equal to about 370 ng/ml in the blood. Although cort levels vary with the assay, we find that acute restraint stress elicits a cort level of up to 600 ng/ml, such that the 1-uM cort (or Dex) is well within physiological limits, at least with respect to circulating levels in the blood. What final concentration is reached in the highly vascularized PVN is not currently known.

6. I do not understand the graphs in Figs 3G, 4E and 5E. How were the error bars computed? Why do the mean values (15-20 mV) seem so much greater than the shifts shown in the corresponding graphs (5-10mV)?

The shifts shown in the bar graphs were meant to show percent change from resting potential, which was a different, but admittedly confusing, way of expressing the changes in half-activation potential. We have dropped these graphs from the figures.

7. There is a typo in the figure legends: SR141716 is rimonobant not mifepristone

We thank the reviewer for catching this error, which has been corrected.
